# Temporal transcriptome changes induced by MDV in marek's disease-resistant and -susceptible inbred chickens

**DOI:** 10.1186/1471-2164-12-501

**Published:** 2011-10-12

**Authors:** Ying Yu, Juan Luo, Apratim Mitra, Shuang Chang, Fei Tian, Huanmin Zhang, Ping Yuan, Huaijun Zhou, Jiuzhou Song

**Affiliations:** 1 Department of Animal & Avian Sciences, University of Maryland, College Park, MD 20742, USA; 2 USDA, ARS, Avian Disease and Oncology Laboratory, East Lansing, MI 48823, USA; 3 Department of Animal Breeding and Genetics, College of Animal Sciences, China Agricultural University, Beijing, 100193, P.R. China; 4 Department of Poultry Science, TAMU, College Station, 77843-2472, TX

## Abstract

**Background:**

Marek's disease (MD) is a lymphoproliferative disease in chickens caused by Marek's disease virus (MDV) and characterized by T cell lymphoma and infiltration of lymphoid cells into various organs such as liver, spleen, peripheral nerves and muscle. Resistance to MD and disease risk have long been thought to be influenced both by genetic and environmental factors, the combination of which contributes to the observed outcome in an individual. We hypothesize that after MDV infection, genes related to MD-resistance or -susceptibility may exhibit different trends in transcriptional activity in chicken lines having a varying degree of resistance to MD.

**Results:**

In order to study the mechanisms of resistance and susceptibility to MD, we performed genome-wide temporal expression analysis in spleen tissues from MD-resistant line 6_3_, susceptible line 7_2 _and recombinant congenic strain M (RCS-M) that has a phenotype intermediate between lines 6_3 _and 7_2 _after MDV infection. Three time points of the MDV life cycle in chicken were selected for study: 5 days post infection (dpi), 10dpi and 21dpi, representing the early cytolytic, latent and late cytolytic stages, respectively. We observed similar gene expression profiles at the three time points in line 6_3 _and RCS-M chickens that are both different from line 7_2_. Pathway analysis using Ingenuity Pathway Analysis (IPA) showed that MDV can broadly influence the chickens irrespective of whether they are resistant or susceptible to MD. However, some pathways like cardiac arrhythmia and cardiovascular disease were found to be affected only in line 7_2_; while some networks related to cell-mediated immune response and antigen presentation were enriched only in line 6_3 _and RCS-M. We identified 78 and 30 candidate genes associated with MD resistance, at 10 and 21dpi respectively, by considering genes having the same trend of expression change after MDV infection in lines 6_3 _and RCS-M. On the other hand, by considering genes with the same trend of expression change after MDV infection in lines 7_2 _and RCS-M, we identified 78 and 43 genes at 10 and 21dpi, respectively, which may be associated with MD-susceptibility.

**Conclusions:**

By testing temporal transcriptome changes using three representative chicken lines with different resistance to MD, we identified 108 candidate genes for MD-resistance and 121 candidate genes for MD-susceptibility over the three time points. Genes included in our resistance or susceptibility genes lists that are also involved in more than 5 biofunctions, such as *CD8α*, *IL8*, *USP18*, and *CTLA4*, are considered to be important genes involved in MD-resistance or -susceptibility. We were also able to identify several biofunctions related with immune response that we believe play an important role in MD-resistance.

## Background

MD is a serious lymphoproliferative disease in chickens caused by MDV and characterized by transformation of T cells that cause tumors in various organs including liver, spleen, gonads, heart, peripheral nerves, skin and muscle [1-3]. Chickens with MD exhibit over-expression of Hodgkin's disease antigen CD30 (CD30^hi^) that makes it a natural model for studying the initiation and progression of CD30^hi ^lymphomas [4]. MDV is an alphaherpesvirus belonging to the *Mardivirus *genus which contains three members: MDV-1, MDV-2 and HVT (herpesvirus of turkeys) [5-7]. According to Calnek *et al*. [8,9], MDV, like other herpesviruses, goes through a complex life cycle that includes cytolytic and latent phases in host cells. An early cytolytic infection is started at 2-7dpi characterized by the virus particles expressing large amounts of the early protein pp38. Subsequently, a latent phase is initiated at around 7-10dpi with the MDV genome persisting in the host cells. Following latency, a late cytolytic phase causes inflammation and transformation of latently infected lymphocytes into tumor cells and is triggered between 14-21dpi [8,9]. During the first cytolytic phase, MDV first uses B cells as a target for its replication before targeting activated CD4^+ ^T cells to enable a persistent latent infection [10-12].

Two highly inbred chicken lines 6_3 _and 7_2_, sub-lines of lines 6 and 7, have been bred since 1939 with line 6_3 _chickens resistant to MD and line 7_2 _chickens susceptible to MD [13]. To better understand the mechanisms underlying MD-resistance and -susceptibility, several studies have been made to ascertain the differences between these two chicken lines. A much higher virus copy number was observed in line 7 chickens indicating varying levels of virus replication [14]. Different proportions of CD4^+ ^T cells and CD8^+ ^T cells were found in MD-resistant and -susceptible chickens when infected by MDV. In MD-susceptible birds, as the CD4^+ ^T cells increased in number, the number of CD8^+ ^T cells decreased; the opposite occurred in MD-resistant chickens [15]. Lymphocyte surface markers such as Ly-4, Bu-1 and Th-1, were present in different levels in these two chicken lines [16,17]. The expression of some cytokines, such as IL6 and IL18, was also found to differ between line 6 and line 7 chickens[18]. From an epigenetic perspective, differences in promoter DNA methylation levels between line 6_3 _and line 7_2 _chickens have been found in several candidate genes [19].

With the development of functional genomics technologies, some progress has been made towards investigating the mechanism of MD-resistance in a genome-wide manner. Quantitative trait loci associated with MD-resistance or -susceptibility have been mapped to chromosomes 1, 5, 7, 9, 15, 18, 26, Z, E21 and E16 [20-23]. Also, through the use of chicken immune-specific microarrays, immunoglobulin genes have been shown to have a higher expression in MD-resistant chicken lines as compared to MD-susceptible chicken lines [24]. However, the exact mechanisms behind resistance and susceptibility to MD are still unknown.

Researchers at the Avian Disease and Oncology Laboratory (ADOL, East Lansing, MI, USA) have developed nineteen recombinant congenic strains (RCS) with varying phenotypic traits from lines 6_3 _and 7_2 _to further investigate the mechanisms of MD-resistance and - susceptibility [13,25]. One of these strains, RCS-M, which was developed from line 6_3 _and line 7_2 _and possesses around 87% genetic background of line 6_3_, is genetically closer to the resistant line 6_3_. Our previous study of tumor incidence rates induced by a partially attenuated very virulent plus (vv+) strain of MDV (648A, passage 40) in the different RCSs revealed that while only 0-3% of line 6_3 _chickens and up to 100% of the line 7_2 _chickens developed tumors after MDV infection, about 40% of RCS-M chickens developed tumors (Data not shown). Because of this intermediate response of RCS-M to MDV infection, it is suitable for us to use these three chicken lines to investigate the mechanism of MD-resistance and susceptibility. We performed a temporal trancriptome analysis with spleen samples from line 6_3_, 7_2 _and RCS-M chickens before and after MDV infection at 5dpi, 10dpi and 21dpi. Our main objective is to build on the current understanding of Marek's disease pathogenesis and immune response to MDV, and this genome-wide approach is used for this purpose. To our knowledge, this is the first comprehensive study combining a chicken line having intermediate resistance to MD together with highly-resistant and susceptible lines to more precisely identify the possible genetic factors behind MD resistance and susceptibility.

## Results

### Temporal Gene Expression Profiles of line 6_3_, line 7_2 _and line M chickens in MDV Challenge Experiment

To find genes that may be involved in the MD-resistance and -susceptibility, we performed transcriptome analysis using three chicken lines with different phenotypes after MDV infection to find the host genes with different reactions to virus infection. We chose 5dpi, 10dpi and 21dpi to represent the critical phases of virus progression to study gene expression changes induced in the different chicken lines.

**Figure 1:**
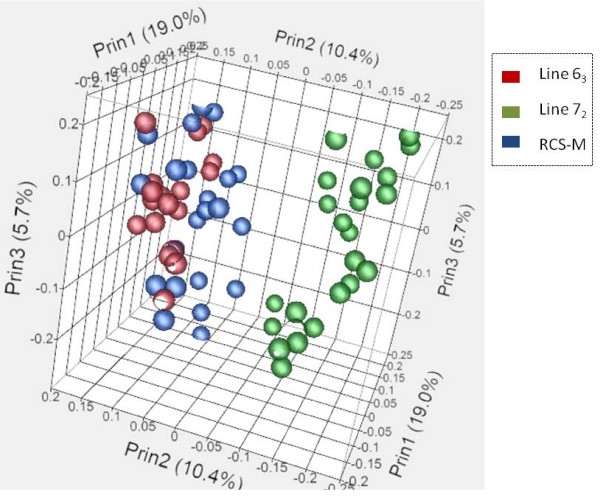
**A three-dimensional PCA plot of 64 individuals indicating broad transcriptional similarities between line 6_3 _and RCS-M that are both markedly distinct from line 7_2_**.

We conducted an initial quality assessment of our dataset to remove outliers (see Methods). Principal component analysis (PCA) was used to compare the global gene expression profiles of these three chicken lines. The preliminary PCA plot indicated broad differences between the three chicken lines with lines 6_3 _and RCS-M clustering together and distinct from line 7_2 _chickens (Figure 1). Data normalization and differential gene expression analysis was performed using the limma package in R (for details see Methods). In order to minimize transcriptional variations owing to growth and other developmental differences between the three chicken lines, individuals were paired by line and dpi and comparisons carried out between infected and non-infected individuals. The p-values were then corrected for multiple comparisons using the Benjamini-Hochberg FDR calculation procedure [26]. To detect the host response to MDV infection, we compared the gene expression level of the infected samples to non-infected control samples at the same time point. Differentially expressed gene lists were obtained using a criteria of P < 0.05, FDR < 0.5 and |logFC| > 1.5.

Our significant gene lists consisted of a total of 11779 genes in the three chicken lines across three time points that included some virus genes contained in the microarray (Additional file 1. Table S1). As our focus was on the host response to MDV infection, we excluded the MDV genes from further analysis reducing our total gene number to 11694 (Table 1). Notably, no gene was found differentially expressed in line 7_2 _at 5dpi using the above criteria. However, when validating the microarray results with qPCR, we found several genes significantly changed by MDV infection in line 7_2 _at 5dpi (Additional file 2. Figure S1).

**Table 1:** Number of genes differentially expressed after MDV infection

	**line 6_3 _(Inf. Vs. Non.)**		**line 7_2 _(Inf. Vs. Non.)**		**RCS-M (Inf. Vs. Non.)**	
	
	**+**	**-**	**+**	**-**	**+**	**-**
	
**5dpi**	777	651	0	0	707	680
**10dpi**	708	660	823	691	791	572
**21dpi**	573	585	1007	1088	567	814

Genes with differential expression were termed with P < 0.05, |LogFC|>1.5 and FDR < 0.5. +: up-regulated after MDV infection; -: down-regulated after MDV infection.

### Pathway analysis to find the networks and biofunctions involved in MDV infection

To study the networks and biofunctions enriched in the differentially expressed genes after MDV infection, we used the IPA system to analyze genes sets. We first used the raw probe names from the microarray as the input data set and found less than 25% of the probes were compatible with IPA. Therefore, we used data mining to map the probe names to homologs from other species that could be used by IPA (for details see Methods). The mapped homologs of these ESTs are shown in Additional file 3. Table S2.

Detailed analyses of the networks and biofunctions affected by MDV in the different chicken lines across different time points were performed to understand the host responses to MDV infection. The top 5 networks influenced by MDV infection in each chicken line at three time points are shown in Table 2. From these networks we can see that during various stages of the MDV life cycle (5dpi, 10dpi and 21dpi), the virus has a broad influence on host gene expression in all chicken lines. A large number of genes involved in metabolism, tissue development, gene expression and the cell cycle were changed by MDV infection in all chicken lines which indicated broad similarities in the host response to MDV infection. However, we also found some unique networks among these chicken lines, such as, cardiac arrhythmia and cardiovascular disease related networks found only in line 7_2_. On the other hand, some immune-related networks such as cell-mediated immune response and antigen presentation were only found in line 6_3 _and RCS-M but not in line 7_2_. These are the specific responses to MDV infection which may be related to the genetic basis of MD-resistance and -susceptibility.

**Table 2:** Enriched networks at different time points of MDV infection in different chicken lines

Lines	Time points	Score	Focus Molecules	Top Functions
L6_3_	5dpi	46	29	Digestive System Development and Function, Hepatic System Development and Function, Organ Development
		36	25	Skeletal and Muscular System Development and Function, Tissue Morphology, Lipid Metabolism
		35	25	Amino Acid Metabolism, Small Molecule Biochemistry, Cellular Compromise
		28	21	Carbohydrate Metabolism, Lipid Metabolism, Small Molecule Biochemistry
		24	20	Drug Metabolism, Endocrine System Development and Function, Lipid Metabolism
	10dpi	36	25	Lipid Metabolism, Small Molecule Biochemistry, Vitamin and Mineral Metabolism
		30	21	Infection Mechanism, Visual System Development and Function, Dermatological Diseases and Conditions
		30	21	Molecular Transport, Cellular Movement, Cell Cycle
		27	22	Neurological Disease, Carbohydrate Metabolism, Lipid Metabolism
		26	19	Organismal Injury and Abnormalities, Infection Mechanism, Infectious Disease
	21dpi	39	28	Cell Morphology, Cellular Function and Maintenance, Cell Death
		34	22	Cardiovascular System Development and Function, Organismal Development, Tissue Development
		27	19	Carbohydrate Metabolism, Small Molecule Biochemistry, Cellular Assembly and Organization
		24	17	Cell-To-Cell Signaling and Interaction, Cell-mediated Immune Response, Cellular Development
		22	16	Lipid Metabolism, Small Molecule Biochemistry, Behavior

L7_2_	10dpi	29	21	Cardiac Arrythmia, Cardiovascular Disease, Genetic Disorder
		27	22	Cellular Assembly and Organization, Cellular Compromise, Free Radical Scavenging
		26	19	Cell-To-Cell Signaling and Interaction, Cellular Growth and Proliferation, Skeletal and Muscular System Development and Function
		24	18	Cell Morphology, Connective Tissue Development and Function, Skeletal and Muscular System Development and Function
		22	17	Infection Mechanism, Cardiovascular System Development and Function, Organismal Development
	21dpi	38	27	Cell Morphology, Connective Tissue Development and Function, Skeletal and Muscular System Development and Function
		32	24	Lipid Metabolism, Molecular Transport, Small Molecule Biochemistry
		31	24	Embryonic Development, Tissue Development, Cell Cycle
		31	24	Cell Morphology, Skeletal and Muscular System Development and Function, Connective Tissue Development and Function
		23	20	Connective Tissue Disorders, Genetic Disorder, Cellular Assembly and Organization

RCS- M	5dpi	32	22	Cell Morphology, Cellular Development, Cell Death
		32	22	Cellular Assembly and Organization, Cell Morphology, Cellular Development
		30	21	Cell Morphology, Cellular Development, Skeletal and Muscular System
				Development and Function
		28	20	Amino Acid Metabolism, Small Molecule Biochemistry, Drug Metabolism
		24	18	Inflammatory Disease, Renal and Urological Disease, Amino Acid Metabolism
	10dpi	34	23	Carbohydrate Metabolism, Lipid Metabolism, Small Molecule Biochemistry
		31	21	Antigen Presentation, Cell-To-Cell Signaling and Interaction, Cellular Function and Maintenance
		29	20	Molecular Transport, Drug Metabolism, Lipid Metabolism
		21	16	Carbohydrate Metabolism, Drug Metabolism, Small Molecule Biochemistry
		20	16	Dermatological Diseases and Conditions, Genetic Disorder, Immunological Disease
	21dpi	33	23	Cell Morphology, Cellular Assembly and Organization, Cell-To-Cell Signaling and Interaction
		26	19	Connective Tissue Development and Function, Skeletal and Muscular System Development and Function, Tissue Morphology
		24	18	Carbohydrate Metabolism, Small Molecule Biochemistry, Organismal Functions
		24	23	Cell Death, Gene Expression, Cellular Function and Maintenance
		23	18	Lipid Metabolism, Molecular Transport, Small Molecule Biochemistry

*The genes input in IPA software were obtained using P < 0.05, |LogFC|>1.5 and FDR<0.5.

### Identification of genes related with MD-resistance and -susceptibility

**Figure 2:**
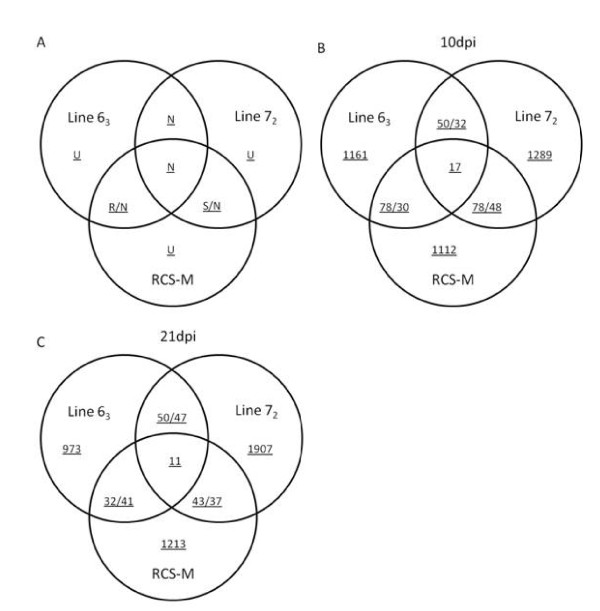
**Venn diagram of the differentially expressed genes after MDV infection in different chicken lines at three time points showing the number of genes that are related to MD-resistance and -susceptibility**. A. Schema showing the gene sets related to MD-resistance and-susceptibility. R: genes related to MD-resistance; S: genes related to MD-susceptibility; U: line specific genes; N: genes with no definition. B. 10 days post infection. C. 21 days post infection. Line 6_3_.non: non-infected control of line 6_3 _chickens; Line 63.inf: infected line 6_3 _chickens; Line 7_2_.non: non-infected control of line 7_2 _chickens; Line 72.inf: infected line 7_2 _chickens; RCS-M.non: non-infected control of RCS-M; RCS M.inf: infected RCS-M chicken.

Utilizing the varying characteristics of these chicken lines, we attempted to identify genes associated with MD-resistance and -susceptibility from pair-wise comparisons. We make the following observations about differentially expressed genes obtained from our analysis. Genes differentially expressed after MDV infection and having similar trends in line 6_3 _and RCS-M but not in line 7_2 _are likely to be related to MD-resistance; conversely, genes showing similar trends in line 7_2 _and RCS-M but not in line 6_3 _are possibly related to MD-susceptibility (Figure 2A). Genes differentially expressed after MDV infection in all three chicken lines are likely indicators of a common host response to virus infection. Finally, genes that are differentially expressed only in one chicken line could be part of a line-specific host response to virus infection (Figure 2A).

From the above intuition, we were able to narrow the list of putative genes contributing to resistance (resistant genes) to 78 and 30 and the number of genes possibly associated with susceptibility (susceptible genes) to 78 and 43 at 10dpi, 21dpi respectively (Figure 2B-D). For some of the putative resistant genes, the fold change after MDV infection in RCS-M is about half or less compared to line 6_3 _suggesting an additive effect of these genes in resistance (Additional file 4. Table S3). We can also find several genes that show a similar behaviour in the susceptible gene lists (Additional file 4. Table S3). This is consistent with the intermediate tumor incidence rates we observed in RCS-M chickens.

Although we were able to limit the number of putative resistant and susceptible genes, it is still a difficult task to determine the most important genes. By further analysing the networks associated with these genes, we found several genes involved in a large number of biofunctions (Additional file 5. Table S4). This indicated the importance of these genes to MD resistance or susceptibility even though they may not have very high fold changes. We defined high-confidence genes as those involved in more than 5 biofunctions to obtain high-confidence gene lists important for MD-resistance or -susceptibility (Table 3). The differentially expressed genes at the various time points were different indicating different mechanisms involved in the host response. At 10 dpi several interleukin genes were present among the putative susceptible genes such as IL8, IL17A and IL12RB2. The NOS2 gene, which can catalyze the generation of NO (nitric oxide), was also found in the putative MD-susceptible gene list.

**Table 3:** Genes from MD-resistant and -susceptible gene lists enriched in more than 5 biofunctions

T.Point	R./S.	Genes	+/-	No. Bio.	P.Name	S. Name	Description
10dpi	R.	GNAQ	-	39	*002455	AJ851735	Q5F3B5 : (Q5F3B5) Hypothetical protein
	
	R.	PRKG1	-	33	*015981	BU421057	Q9Z0Z0 : (Q9Z0Z0) cGMP-dependent protein kinase 1, beta isozyme (CGK 1 beta) (cGKI-beta)
	
	R.	CD8A	+	27	*007686	CR390735	XP_420863 : gi:50747402:ref:XP_420863.1: PREDICTED: similar to CD8 alpha chain [Gallus gallus]
	
	R.	RGS4	-	27	A_87_P021537	BX950639	Gallus gallus finished cDNA, clone ChEST606e8. [BX950639]
	
	R.	RARB	-	25	A_87_P008918	X56674	Chicken mRNA for retinoic acid binding protein beta isoform. [X56674]
	
	R.	HAS3	+	12	*013355	BU278152	Q8CEB9 : (Q8CEB9) Mus musculus 10 days neonate skin cDNA, RIKEN full-length enriched library, clone:4732404L04 product:similar to DG42III
	
	R.	SPINK5	-	12	*005267	CR353088	P10184 : gi:1708509:sp:P10184:IOV7_CHICK Ovoinhibitor precursor &gt;gnl:BL_ORD_ID:146450 gi:212485:gb:AAA48994.1: ovoinhibitor
	
	R.	SULF2	-	10	A_87_P022688	BX934216	Gallus gallus finished cDNA, clone ChEST442e20. [BX934216]
	
	R.	COL14A1	-	9	A_87_P008851	X70793	G.domesticus mRNA for collagen XIV (longer splice variant). [X70793]
	
	R.	SCN4B	-	9	A_87_P021873	BX935954	Gallus gallus finished cDNA, clone ChEST430l23. [BX935954]
	
	R.	WISP1	-	9	A_87_P009711	DQ003338	Gallus gallus WNT1 inducible signaling pathway protein 1 (WISP-1) mRNA, complete cds. [DQ003338]
	
	R.	AHNAK	-	5	*004305	BX935083	NP_033773 : gi:61743961:ref:NP_033773.1: AHNAK nucleoprotein isoform 1 [Mus musculus]
	
	R.	P4HA2	-	5	A_87_P007103	TC198023	Q8BU53 (Q8BU53) Mus musculus 2 days pregnant adult female oviduct cDNA, RIKEN full-length enriched library, clone:E230038K10 product:procollagen-proline, 2-oxoglutarate 4-dioxygenase (proline 4-hydroxylase), alpha II polypeptide, full insert sequence, p
	
	S.	NOS2	+	64	A_87_P009073	U46504	Chicken macrophage nitric oxide synthase mRNA. [U46504]
	
	S.	IL8	-	58	*010230	Y14971	O73912 : (O73912) K60 protein precursor (CXC chemokine K60)
	
	S.	IL17A	+	53	A_87_P035017	AY920750	Gallus gallus interleukin 17 mRNA, complete cds. [AY920750]
	
	S.	CTLA4	+	47	*019882	DN853042	XP_421960 : gi:50750341:ref:XP_421960.1: PREDICTED: similar to costimulatory molecule B7 receptor CD152 [Gallus gallus]
	
	S.	IL12RB2	+	23	*000640	AJ621939	Q5GR16 : (Q5GR16) Interleukin 12 receptor beta 2 (Fragment)
	
	S.	PLK1	+	20	*001748	AJ720598	Q5ZJ36 : (Q5ZJ36) Hypothetical protein
	
	S.	ROR1	-	13	*000634	AJ620298	Q705C2 : (Q705C2) Tyrosine kinase orphan receptor 1
	
	S.	LECT2	-	11	*009802	M29449	O88803 : (O88803) Leukocyte cell-derived chemotaxin 2 precursor (Chondromodulin II) (ChM-II)
	
	S.	ABCA8	-	6	*016305	BU440826	XP_415691 : gi:50757881:ref:XP_415691.1: PREDICTED: similar to ATP-binding cassette, sub-family A, member 10; ATP-binding cassette A10 [Gallus gallus]
	
	S.	TSPAN8	-	6	*004228	BX934864	XP_416096 : gi:50728338:ref:XP_416096.1: PREDICTED: similar to Tm4sf3 protein [Gallus gallus]
	
	S.	COL5A2	-	6	A_87_P001489	TC224310	Q86XF6 (Q86XF6) COL5A2 protein, partial (5%) [TC224310]
	
	R.	MMP9	-	63	A_87_P037769	AF222690	Gallus gallus 75 kDa gelatinase mRNA, complete cds. [AF222690]

21dpi	R.	SFTPA1	+	38	*000489	AF411083	Q90XB2 : (Q90XB2) Surfactant protein A precursor
	
	R.	FBXW4	-	6	*015952	BU419266	Q9JMJ2 : (Q9JMJ2) F-box/WD-repeat protein 4 (F-box and WD-40 domain protein 4) (Hagoromo protein)
	
	R.	CPN1	-	5	*006641	CR387490	Q9EQV8 : (Q9EQV8) Carboxypeptidase N, polypeptide 1, 50kD
	
	S.	CD28	+	50	*010136	X67915	P31043 : (P31043) T-cell-specific surface glycoprotein CD28 homolog precursor (CHT28)
	
	S.	CHRM4	-	37	A_87_P009241	NM_001031191	Gallus gallus cholinergic receptor, muscarinic 4 (CHRM4), mRNA [NM_001031191]
	
	S.	F10	-	29	*009605	D00844	P25155 : (P25155) Coagulation factor × precursor (Stuart factor) (Virus activating protease) (VAP)
	
	S.	WNT7A	-	27	A_87_P038155	AB045629	Gallus gallus mRNA for Wnt-7a, complete cds. [AB045629]
	
	S.	FMN2	+	12	A_87_P005690	TC203559	AF218942 formin 2-like protein {Homo sapiens;}, partial (28%) [TC203559]
	
	S.	ATF6	-	8	A_87_P023703	BX931991	Gallus gallus finished cDNA, clone ChEST222o13. [BX931991]
	
	S.	GLO1	-	6	*004233	BX934880	XP_419481 : gi:50740506:ref:XP_419481.1: PREDICTED: similar to glyoxylase 1; glyoxalase 1 [Gallus gallus]
	
	S.	OPN4	-	5	*002725	AY882944	XP_421494 : gi:50749124:ref:XP_421494.1: PREDICTED: similar to Opsin 4 (Melanopsin) [Gallus gallus]
	
	S.	ITGBL1	-	5	*016603	BU449144	Q4VBJ0 : (Q4VBJ0) Integrin, beta-like 1

T.Point: time point; No. Bio.: number of biofunctions involved; P.Name: Probe Name; S.Name: Systematic Name; R.: resistant genes; S.: susceptible genes; +: up-regulated; -:down-regulated.

### Validation of the microarray results by real-time quantitative PCR

To validate the microarray results, we designed primers for some high-confidence genes such as *CD8α*, *CTLA4, IL8 *and *USP18 *and some other genes chosen at random, such as *CD8β*, *GHR*, *TNFRSF6B *and *MMP2*. Since a reference gene with stable expression is essential to avoid distortions in qPCR, the two genes *GAPDH *and *ACTB *are commonly used as internal reference for doing qPCR of MDV infected samples [24,27]. In our validation, we first used both genes as internal references to see if there were any distortions and found no differences (Additional file 6. Figure S2). Therefore, *GAPDH *was chosen as the internal reference. We also designed primers that span introns to further avoid the influence of DNA contamination. As shown in Additional file 2. Figure S1, we were able to validate most of the genes that are differentially expressed. Also, comparable expression profiles were observed for most of the validated genes in the microarray and qPCR (Table 4) which further suggested that the gene expression profiles from the microarray are reliable.

**Table 4:** Validation of microarray results by quantitative PCR

Genes	Probe Name in Micro-array	Time points	Gene expression fold change after MDV infection in each lines (Inf./Non.)					
			
			line 6_3_		line 7_2_		RCS-M	
			
			Micro- array	Q-PCR	Micro- array	Q-PCR	Micro-Array	Q-PCR
		5dpi	1.02	***1.45***	2.85	***4.07***	1.41	1.29
*CTLA-4*	*019882	10dpi	2.31	***3.37***	***3.29***	***13.68***	***3.56***	***7.02***
		21dpi	0.62	***2.05***	***3.07***	2.03	1.27	1.44

		5dpi	1.46	1.05	1.41	1.19	1.52	0.85
*CD8α*	*007686	10dpi	***2.92***	***2.33***	1.51	1.35	***3.90***	2.80
		21dpi	1.08	***3.13***	***0.35***	***0.05***	1.24	2.01

		5dpi	1.17	1.14	1.07	0.97	1.14	0.78
*CD8β*	A_87_P008699	10dpi	***1.87***	***2.86***	1.03	2.05	***1.86***	2.30
		21dpi	1.42	***3.31***	***0.32***	***0.04***	1.10	***2.03***

		5dpi	1.24	***3.37***	4.09	4.13	1.43	1.68
*USP18*	*005670	10dpi	2.07	2.15	***4.87***	***5.55***	2.53	0.58
		21dpi	0.98	11.89	***3.28***	4.54	***2.73***	***5.99***

		5dpi	1.00	2.85	5.67	***9.36***	1.12	***4.17***
*TNFRSF6B*	*003404	10dpi	***5.03***	***9.47***	***5.52***	***11.46***	2.75	2.35
		21dpi	1.54	***5.27***	***4.28***	***9.14***	1.00	***6.85***

		5dpi	1.00	0.64	0.60	***0.28***	***0.62***	***0.29***
*MMP2*	A_87_P009159	10dpi	0.78	0.89	***0.63***	0.93	***0.54***	0.62
		21dpi	0.88	1.10	***0.22***	***0.02***	0.82	1.16

		5dpi	1.00	0.72	1.04	***0.18***	1.71	0.91
*IL8*	*010230	10dpi	***8.28***	0.66	***3.01***	***0.19***	***4.25***	***0.10***
		21dpi	18.51	2.22	***0.46***	***0.02***	0.81	***2.59***

		5dpi	1.00	***3.14***	1.00	***0.45***	1.85	0.81
*GHR*	A_87_P009190	10dpi	***8.57***	***1.25***	0.24	***1.82***	1.00	***0.53***
		21dpi	1.54	***1.56***	0.40	0.59	1.00	***2.76***

The numbers here represent the fold change of gene expression after MDV infection. The numbers equal to 1.00 means the expression level doesn't change after MDV infection. The numbers > 1.00 means the expression level is increased after MDV infection while numbers < 1.00 indicate that the expression level is reduced after MDV infection. The numbers in bold and italic are statistically significant (P < 0.05).

## Discussion

There have been several studies looking at gene expression changes related to disease in general [28-30] and MD in particular [24,27,31-34], although results tend to vary a lot. MD is a complex disease with the disease phenotype in susceptible individuals depending on the location and frequency of tumors. Any single gene with differential expression cannot fully explain the phenomenon of host resistance or susceptibility. Therefore, we tried to use a genome-wide approach to build on the current understanding of Marek's disease pathogenesis and immune response to MDV. Upon close examination of the transcriptional responses, dramatically increased numbers of significant genes were observed at 10dpi in RCS-M and at 21dpi in line 7_2 _at lower FDR levels (FDR < 0.2) which indicated a strongly enhanced transcriptional response. At a more relaxed FDR level (FDR < 0.5), we find comparable numbers of differentially expressed genes at 5, 10 and 21dpi in line 6_3 _and RCS-M. Line 7_2 _has similar numbers of significant genes at 10dpi but there is a definite increase in the transcriptional response at 21dpi with close to twice as many differentially expressed genes. However, even at this level, we do not find any significantly expressed genes at 5dpi in line 7_2 _(FDR<0.5), indicating a much muted transcriptional response (Table 1).

Over the years, several attempts have been made to identify the gene profiles that change as a result of MDV infection. For example, using microarray analysis, studies have identified some genes related to MDV infection by using different chicken lines and MDV strains [24,31-34]. When chicken embryo fibroblasts were infected with MDV, genes related to inflammation, cell-growth and differentiation and antigen presentation, such as *MIP*, *IL-13R*, *MHC I *and *MHC II *were induced both at 2dpi and 4dpi [33]. In contrast, in spleen tissue, several other genes were found to be affected at an early stage, including *TLR-15*, *IL-6 *and *Mx1*[31]. In chickens with major histocompatibility complex (MHC)-associated MD resistance, the immunoglobulin genes *IgG *and *IgM *were differentially expressed after MDV infection at 7dpi and 14dpi[24], whereas in lines 6 and 7 from ADOL, that carry the same MHC haplotype (B^2^) but differ in their response to MDV infection[35], various alloantigens like Ly-4 [16] and Bu-1 [17] were differentially expressed. Linkage and association studies as well as integrated analyses using genetic mapping and microarrays have revealed some genes that may be responsible for MD progression or resistance, such as *GH*, *IFNγ *and *SULT *[27]. However, it is difficult to find a consensus amongst these studies due to variation in experimental parameters such as, virus strain or *in vitro *derived samples. By using a genome-wide approach and three chicken lines with varying resistance to MD, we were able to generate a comprehensive list of candidate genes that can be used for studying MD-resistance and susceptibility. Besides finding some genes that were reported in previous studies, such as *Mx1*, we also found several genes that have not been reported before in this context, such as *CD8α, IL8, USP18*, and *CTLA4*. *CD8α*, present in the putative resistant gene list at 10dpi, codes a surface glycoprotein expressed on a subpopulation of cytotoxic T lymphocytes (CTLs) [36], which binds to the α3 domain or membrane-proximal domain of most of the known HLA class I molecules to enhance CTL activation [37-41]. It has been shown that *CD8α *was up-regulated by MDV infection at the early cytolytic stage (4dpi and 7dpi), whereas *IgM *and *CD3 *were down-regulated [34]. These are similar to our microarray results, the slight difference being possibly due to the differences in virus strains and genetic background of the chickens. The *CD8α *gene was significantly up-regulated at 10dpi in the MD-resistant chicken line (line 6_3_) and RCS-M, but down-regulated in the MD-susceptible chicken line (line 7_2_). In chickens vaccinated against MDV an increase of CD8α cells was found after MDV infection compared to unvaccinated chickens [32]. The vaccinated birds were phenotypically similar to line 6_3 _and hence, this result is consistent with our finding. The above evidence, taken all together, leads us to speculate that *CD8α *plays an important role in MD resistance. The induction of *CD8α *gene may result from an increase of the CD8^+ ^T cells that eliminate MDV infected cells in the resistant chickens. However, this scenario needs further validation.

In contrast, CTL-associated antigen-4 (*CTLA-4*), present in our putative susceptible gene list at 10dpi, is a member of the immunoglobulin superfamily expressed on the surface of an activated T cell [42]. It has been reported that the knockout of *CTLA-4 *resulted in a lymphoproliferative disorder and death in mice, which indicated a very important role of CTLA-4 in negative regulation of T cell activation [43]. The blockade of the CTLA-4 pathway results in a rejection of tumor [44,45], indicating that a lower *CTLA-4 *expression may be important for antitumor response. Therefore, in humans, a current strategy of immunotherapy focuses on the blockade of the *CTLA-4 *pathway [46,47]. A higher expression of *CTLA-4 *was detected at lymphoma lesions in MD-susceptible chickens at 21dpi, although no significant difference was found in the whole tissue [48]. Importantly, a similar result existed in our data: the fold change of *CTLA-4 *at 10dpi after MDV infection is much less in line 6_3 _than in line 7_2 _and RCS-M (Table 4), indicating a lower level of *CTLA-4 *involved in antitumor immune response.

In addition to the above genes, some networks and biofunctions were also observed to be different between MD-resistant and susceptible chickens. It is interesting to note that most of the differentially expressed genes were not enriched in biofunctions of immune related diseases, but with other diseases or metabolism. This is consistent with the fact that apart from the generation of tumors, MD-susceptible chickens also exhibit weight loss, paralysis and other symptoms. However, some immune response-related biofunctions were enriched only in line 6_3 _and RCS-M chickens. It was thought for a long time that tumor cells have no antigen and this enables them to escape the host immune system. While the finding of the melanoma antigen in the late 1980's shed light on the role of immune system to fight against tumors [49], the tumor cells are known to also have immunosuppressive agents that help them evade detection and killing by the immune system [50,51]. The networks related to immune response found in line 6_3 _chickens, suggests that in these chickens the immune system is activated to counteract the development of tumor. In contrast, the transformed cells in susceptible chickens are able to escape the natural resistance of the immune system to generate tumors, although at present it is still unclear if this is due to a larger initial damage to the immune system [52] or the immunosuppression induced by MDV in line 7_2 _chickens. NOS2 is an enzyme that catalyzes the generation of NO [53] which in turn increases the virulence of MDV by immunosuppression [54]. However, it has also been shown that NO has inhibitory effects on MDV replication [55] and NO production in MDV-infected susceptible chickens (MHC, B^13^B^13^) is the lowest in comparison to MD-infected resistant birds (MHC, B^19^B^19^) [55,56]. Interestingly, we found that the *NOS2 *gene was up-regulated in susceptible chicken lines. Therefore, it remains to be seen whether the up-regulation of *NOS2 *in line 7_2 _could induce immunosuppression and increase the risk of tumor generation in MD-susceptible chickens. The above results indicate that different immune response in resistant and susceptible chickens lead to the vastly different responses to MDV infection.

To minimize transcriptional variations and take full advantage of a similar genetic background in the inbred lines, we paired birds by line and dpi, respectively, and tested the difference between infected and non-infected individuals. This procedure not only led us to identify the genes most likely related to MD resistance and susceptibility, but also revealed a common broad influence of MDV infection on the hosts. By using IPA to analyze differentially expressed gene sets, we found focused pathways enriched in metabolism, tissue development, gene expression and cell cycle along with other immune-related pathways preferentially enriched in resistant chickens. These results suggested possible mechanisms and specific genes related to MD-resistance or -susceptibility. We hypothesized that there are four possible causes behind MD-resistance: some genes activated in resistant chickens can (i) cause loss of the MDV receptor, (ii) help to clear infected cells, (iii) affect the viral life cycle or (iv) prevent transformation of infected cells. Our observations and previous research have showed that the virus load in both resistant and susceptible chickens was similar at early stages of infection [18,57], suggesting the presence of receptors for MDV in both resistant and susceptible chickens. Thus, the latter three of the aforementioned possibilities are more likely to be the main reasons for MD-resistance although it is not easy to say which of these play a big role in non-MHC associated resistance.

## Conclusions

Using a comprehensive genome-wide study of gene expression in chicken lines with varying resistance to MD, we were able to identify pathways and genes that may be involved in MD-resistance and susceptibility. Phenotypic similarities between the chicken lines enabled us to narrow the list of putative genes to 108 genes associated with MD-resistance and 121 genes associated with susceptibility. Combining network analysis with differential gene expression analysis helped uncover high-confidence genes such as *CD8α*, *IL8*, *USP18*, and *CTLA-4 *and several immune-related biofunctions with potentially important consequences to MDV infection. Our findings add to the current understanding of the mechanism behind resistance and susceptibility to MD while expanding the scope of future studies with a comprehensive list of putative genes. Our approach also underlines the importance of comprehensive functional studies to gain valuable biological insight into the genetic factors behind complex disease.

## Methods

### Sample Collection for Microarray

Three inbred lines of White Leghorn (line 6_3_, line 7_2 _and RCS-M) were divided into two treatment groups each containing 60 chickens. One group from each line was infected with a partially attenuated very virulent strain (vv^+^) of MDV-648A passage 40 [1], at day 5 after hatch, intra-abdominally at a viral dosage of 500 plaque-forming units (PFU) per chick. The other group was not infected. The viral-challenge experiment was conducted in the BL-2 facility at ADOL. Four chickens from each group were euthanized at 5dpi (cytolytic infection period), 10dpi (latency period) and 21dpi (reactivation period), respectively. Spleen samples were collected and stored in RNAlater solution (Qiagen, Valencia, CA, USA) at -20°C until RNA extraction. All the experimental chickens were managed and euthanized following ADOL's Guidelines for Animal Care and Use (revised April, 2005) and the Guide for the Care and Use of Laboratory Animals published by Institute for Laboratory Animal Research (ILAR Guide) in 1996 (http://www.nap.edu/openbook.php?record_id=5140).

### RNA Extraction

Approximately 30~50mg spleen tissues were homogenized in TRizol Reagent (Invitrogen, Frederick, MD, USA), and total RNA extraction was performed according to the manufacturer's instructions (Invitrogen, Frederick, MD, USA). Total RNA was purified using the RNAeasy mini column (Qiagen, Valencia, CA, USA) and contaminant DNA was digested by DNase I (Qiagen, Valencia, CA, USA). RNA concentration was assessed using Nanodrop ND-1000 spectrophotometer (Thermo Scientific, Wilmington, DE, USA) and RNA quality determined by 2100 Bioanalyzer (Agilent, Foster City, CA, USA).

### Microarray Experiment Design, Hybridization and Analysis

Custom Agilent 4 × 44K chicken microarrays were used in this study. The 4 × 44K chicken arrays were designed based on the whole chicken genome sequence and consist of 42,034 probes [58]. Four biological replicates of each group were carried out at each time point. RNA was labelled using the Agilent Quick-Amp labelling kit (Agilent Technologies, Santa Clara, CA, USA). In two of the four replicates of each experimental group, the infected samples were labelled with Cy3 and the uninfected two were labelled with Cy5. A total of 825 ng of Cy3 and Cy5 labelled cDNAs were then hybridized to the 4 × 44K Agilent chicken arrays. Following stringency washes, slides were scanned on an Agilent G2505B microarray scanner and the resulting image files analyzed with Agilent Feature Extraction software (version 9.5.1). All procedures were carried out according to the manufacturer's protocols. After the microarray analysis was performed in three chicken lines at three time points, we first tested for the presence of outliers (JMP Genomics, Version 9). Parallel plots and PCA revealed the presence of outliers in our datasets, which were subsequently removed. A parallel plot subsequently indicated that the log_2 _intensities had similar distributions across all remaining arrays (Additional file 7. Figure S3). After the initial quality assessment step we performed linear modelling using the limma package in R to find differentially expressed genes. Dye bias was removed by normalizing within array using loess normalization [59] and normalization between arrays was carried out using quantile normalization [60]. The p-values were corrected for multiple comparisons using the Benjamini-Hochberg FDR calculation procedure [26]. We compared age-matched individuals from the same line before and after infection with MDV at three time points of disease progression. All the array data discussed in this publication have been deposited in NCBI's Gene Expression Omnibus (Yu *et al*., 2010) and are accessible through GEO Series accession number GSE24017 (http://www.ncbi.nlm.nih.gov/geo/query/acc.cgi?acc=GSE24017).

### Data Mining and Network Analysis

The expressed sequence tags (ESTs) specific to microarray probes were mapped to proteins or protein homologs with GenBank names, Swissprot, pfam or RefSeq accession numbers using the BioMart data mining system via Sigenae (Details found on http://www.sigenae.org). Proteins with the identity of 40% or more were considered to be homologs [61]. In case of multiple proteins mapping to a probe, proteins with the highest identity were used to create a unique mapping. The resultant list was then analyzed using IPA to detect the enrichment of biofunctions and networks. Core analysis was performed in IPA using significantly expressed genes from the statistical analysis based on the Ingenuity Knowledge Base with the reference set "Genes + Endogenous Chemicals".

### Quantitative Real-time RT-PCR

RNA samples for quantitative real-time PCR were used for first strand cDNA synthesis using 1 *μg *of total RNA by SuperScript™ II Reverse Transcriptase (Invitrogen, Frederick, MD, USA) with oligo (dT)_12-18 _primers (Invitrogen, Frederick, MD, USA). Samples were then analyzed with real time RT-PCR using an iCycler iQ PCR system (Bio-Rad, Hercules, CA, USA). The real time RT-PCR reactions were performed in a final volume of 20 *μ*l with the QuantiTect SYBR Green PCR Kit (Qiagen, Valencia, CA, USA) according to the manufacturer's instructions. Each group has 4 biological replicates with 3 replicates for one reaction and each reaction was repeated twice. The mRNA expression was normalized against the housekeeping gene *GAPDH *(glyceraldehyde-3-phosphate dehydrogenase). The primers for all the genes analyzed are in Additional file 8. Table S5. All steps of our Q-PCR validation, which include RNA extraction, cDNA synthesis, reference gene selection, Q-PCR procedures, and data analysis were performed according to the *Minimum Information for Publication of Quantitative PCR Experiments *(MIQE) guidelines [62]

## Competing interests

The authors declare that they have no competing interests.

## Authors' contributions

YY extracted RNA, performed array hybridization and partial data analysis. JL performed the pathway analysis, experimental confirmation of microarray results and wrote the paper. AM analyzed the microarray data and wrote the paper. FT extracted RNA. HMZ collected samples and revised the paper. PY did data mining before IPA analysis. JZS designed the experiments and revised the paper. All authors read and approved the final version of this manuscript.

## Supplementary Material

Additional file 1 **Table S1. Number of genes differentially expressed after MDV infection including MDV genes**. This table contains the number of genes that are differentially expressed after MDV infection which including all the genes that were shown in the microarray like MDV genes. Genes with differential expression were termed with p < 0.05, LogFC>1.5 and FDR < 0.5. +: up-regulated after MDV infection; -: down-regulated after MDV infection. Click here for file

Additional file 2 **Figure S1. Validation of microarray data by Q-PCR**. This figure showing the Q-PCR validation result of the genes that shown significant different expression after MDV infection in three time points (5dpi, 10dpi, and 21dpi). Line 6_3_.non: non-infected control of line 6_3 _chickens; Line 63.inf: infected line 6_3 _chickens; Line 7_2_.non: non-infected control of line 7_2 _chickens; Line 72.inf: infected line 7_2 _chickens; non: non-infected control of chicken; inf: infected RCS-M chicken. n = 4 for each line. *P < 0.05, **P < 0.01. Click here for file

Additional file 3 **Table S2. Homologs of chicken ESTs from BioMart**. This table includes the homologs that were converted from the chicken ESTs on the microarray. The data mining was down on BioMark (details see http://www.sigenae.org). Click here for file

Additional file 4 **Table S3. Possible MD-resistant and susceptible gene lists at 10dpi and 21dpi**. This table showed the possible candidates for MD-resistance and susceptibility at 10dpi and 21dpi. The gene lists were chosen by the following criteria: Genes differentially expressed after MDV infection and having similar trends in line 63 and RCS-M but not in line 72 are likely to be related to MD-resistance; conversely, genes showing similar trends in line 72 and RCS-M but not in line 63 are possibly related to MD-susceptibility. Click here for file

Additional file 5 **Table S4. Biofunction enrichment of the MD-resistant and susceptible genes at 10dpi and 21dpi**. The genes that are listed as possible MD-resistant and -susceptible genes were used to do the IPA analysis. This table showed the biofunctions that were enriched by these genes at 10dpi and 21dpi. Click here for file

Additional file 6 **Figure S2. Stability test of the internal reference**. *ACTB *gene was added to normalize the gene expression when doing the Q-PCR experiment to monitor the stability of the Q-PCR result when using *GAPDH *as the internal control. A similar ratio shown in both normalization method indicated a stabilized system of the Q-PCR. Click here for file

Additional file 7 **Figure S3. A parallel plot of kernel densities shows similar distribution of log2 intensities in all arrays after normalization**. Click here for file

Additional file 8 **Table S5. Primers for validation of microarray results by quantitative PCR**. Click here for file
