# Supplementary figures and images for: Temporal transcriptome changes induced by MDV in marek's disease-resistant and -susceptible inbred chickens
Source: BMC Genomics. 2011 Oct 12;12:501. doi: 10.1186/1471-2164-12-501 (PMC3269463; doi:10.1186/1471-2164-12-501)

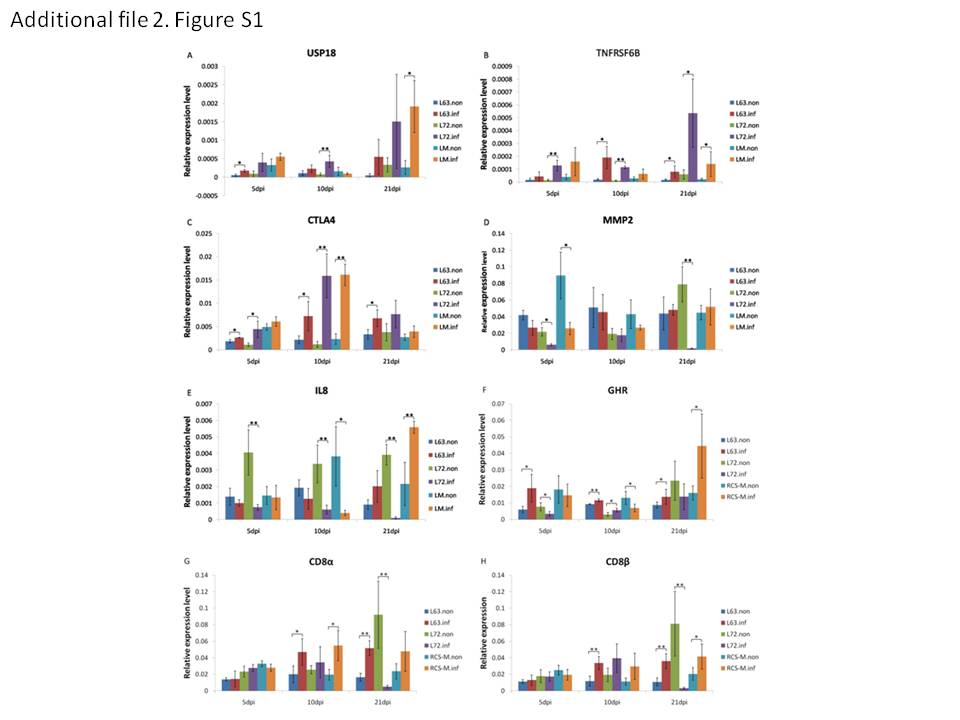

Supplement: Additional file 1 — Table S1. Number of genes differentially expressed after MDV infection including MDV genes. This table contains the number of genes that are differentially expressed after MDV infection which including all the genes that were shown in the microarray like MDV genes. Genes with differential expression were termed with p < 0.05, LogFC>1.5 and FDR < 0.5. +: up-regulated after MDV infection; -: down-regulated after MDV infection. [file 1471-2164-12-501-S1.JPEG]

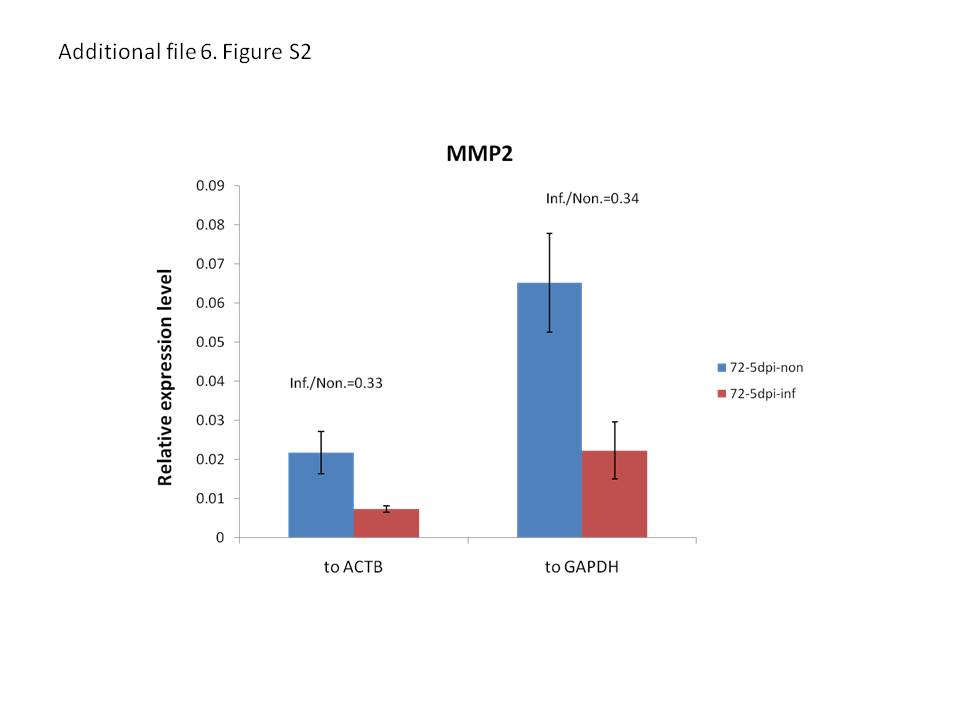

Supplement: Additional file 2 — Figure S1. Validation of microarray data by Q-PCR. This figure showing the Q-PCR validation result of the genes that shown significant different expression after MDV infection in three time points (5dpi, 10dpi, and 21dpi). Line 63.non: non-infected control of line 63 chickens; Line 63.inf: infected line 63 chickens; Line 72.non: non-infected control of line 72 chickens; Line 72.inf: infected line 72 chickens; non: non-infected control of chicken; inf: infected RCS-M chicken. n = 4 for each line. *P < 0.05, **P < 0.01. [file 1471-2164-12-501-S2.JPEG]

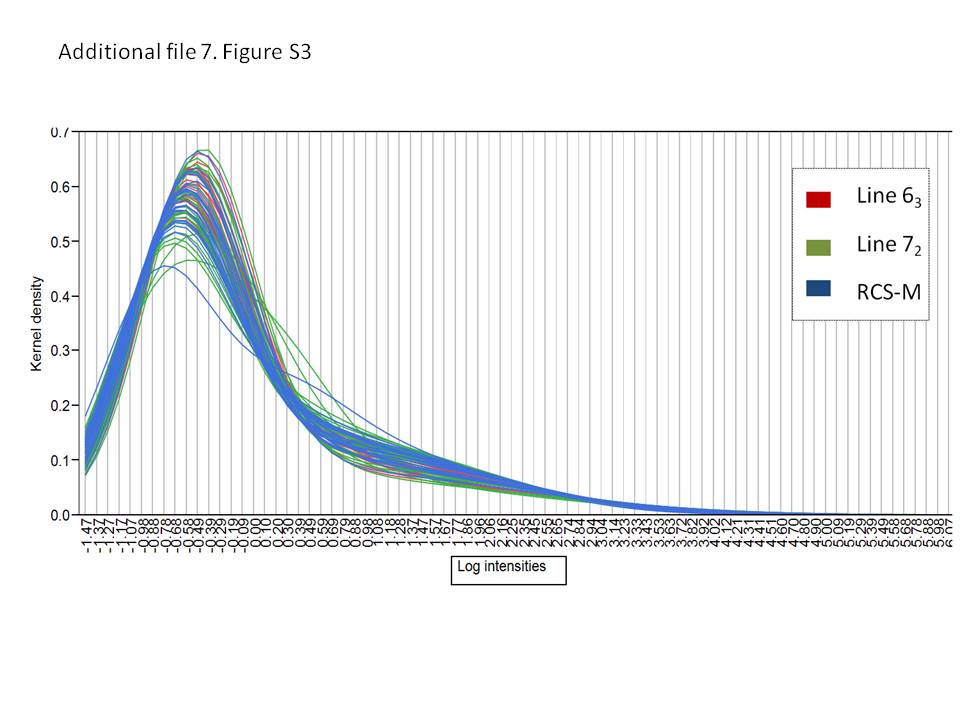

Supplement: Additional file 3 — Table S2. Homologs of chicken ESTs from BioMart. This table includes the homologs that were converted from the chicken ESTs on the microarray. The data mining was down on BioMark (details see http://www.sigenae.org). [file 1471-2164-12-501-S3.JPEG]
