# Supplementary material for: Temporal transcriptome changes induced by MDV in marek's disease-resistant and -susceptible inbred chickens
Source: BMC Genomics. 2011 Oct 12;12:501. doi: 10.1186/1471-2164-12-501 (PMC3269463; doi:10.1186/1471-2164-12-501)
Supplement: Additional file 4 — Table S3. Possible MD-resistant and susceptible gene lists at 10dpi and 21dpi. This table showed the possible candidates for MD-resistance and susceptibility at 10dpi and 21dpi. The gene lists were chosen by the following criteria: Genes differentially expressed after MDV infection and having similar trends in line 63 and RCS-M but not in line 72 are likely to be related to MD-resistance; conversely, genes showing similar trends in line 72 and RCS-M but not in line 63 are possibly related to MD-susceptibility. [file 1471-2164-12-501-S4.DOCX]

Additional file 1. Table S1 Number of genes differentially expressed after MDV infection including MDV genes

|  | **line 6_3_** | | **line 7_2_** | | **RCS-M** | |
| --- | --- | --- | --- | --- | --- | --- |
|  | **+** | **-** | **+** | **-** | **+** | **-** |
| **5dpi** | 779 | 652 | 0 | 0 | 724 | 683 |
| **10dpi** | 711 | 661 | 834 | 693 | 796 | 572 |
| **21dpi** | 574 | 588 | 1035 | 1090 | 570 | 817 |

## Genes with differential expression were termed with p<0.05, ǀLogFCǀ>1.5 and FDR < 0.5. +: up-regulated after MDV infection; -: down-regulated after MDV infection.
