# Supplementary material for: Temporal transcriptome changes induced by MDV in marek's disease-resistant and -susceptible inbred chickens
Source: BMC Genomics. 2011 Oct 12;12:501. doi: 10.1186/1471-2164-12-501 (PMC3269463; doi:10.1186/1471-2164-12-501)
Supplement: Additional file 8 — Table S5. Primers for validation of microarray results by quantitative PCR. [file 1471-2164-12-501-S8.DOCX]

Additional file 8. Table S5 Primers for validation of microarray results by quantitative PCR

| **Genes** | **ProbeName in Microarray** | **GeneBank Accession Number** | **Primers** | ***Sequence*** |
| --- | --- | --- | --- | --- |
| *USP18* | *005670 | XM_416398 | F  R | *5’- GTGGAGCTCAACATCTGCAA-3’*  *5’- CACTTCTTTTGTGGGGGAAA-3’* |
| *TNFRSF6B* | *003404 | XM_417434 | F  R | *5’- GTGCCTCTACTGCAACGTCA-3’*  *5’- ACTGACACGCTCTGTTGTGG-3’* |
| *CTLA-4* | *019882 | NM_001040091 | F  R | *5’- TCAAACAGACAGGCGACAAG-3’*  *5’- GGGCTAACATGGCACTGAAT-3’* |
| *MMP2* | A_87_P009159 | NM_204420 | F  R | *5’- GCTTTCTGCTTAGGCATTGG-3’*  *5’- GCATTGGCATTTCATGTTTG-3’* |
| *CD8α* | *007686 | NM_205235 | F  R | *5’-TTGGACGGGACCTTACAGAC-3’*  *5’-TCAGCCTGCAGGAGTGTAAA-3’* |
| *CD8β* | A_87_P008699 | NM_205247 | F  R | *5’-CTCCTACAGGCTGCACATCA-3’*  *5’-AGAGGCAAAACATCGACCAC-3’* |
| *GHR* | A_87_P009190 | NM_001001293 | F  R | *5’-CTTCAGTGCAAGCGACACAT-3’*  *5’-GGCCATGACTCTCTGCTTTC-3’* |
| *IL8* | *010230 | NM_205498 | F  R | *5’-TGGAGCTGTCCTGGCCCTCC-3’*  *5’ GCCGCTTGGCGTCAGCTTC -3’* |
| *ACTB* |  | NM_205518 | F  R | *5’-TGCGCATAAAACAAGACGAG-3’*  *5’-GACTGCTGCTGACACCTTCA-3’* |
| *GAPDH* |  | NM_204305 | F  R | *5’-GAGGGTAGTGAAGGCTGCTG-3’*  *5’-ACCAGGAAACAAGCTTGACG-3’* |
